# Prevention of febrile neutropenia: use of prophylactic antibiotics

**DOI:** 10.1038/sj.bjc.6605270

**Published:** 2009-09-15

**Authors:** M Cullen, S Baijal

**Affiliations:** 1 University Hospital Birmingham Cancer Centre, Queen Elizabeth Hospital, Edgbaston, Birmingham B15 2TH, UK

**Keywords:** antibiotics, chemotherapy, costs, febrile neutropenia, prophylaxis, resistance

## Abstract

Febrile neutropenia (FN) causes significant morbidity and mortality in patients receiving cytotoxic chemotherapy and can lead to reduced chemotherapy dose intensity and increased overall treatment costs. Antibiotic prophylaxis reduces the incidence of FN. Recent research and meta-analyses confirm that prophylactic fluoroquinolones decrease FN and infection-related mortality in patients with acute leukaemia and those receiving high-dose chemotherapy. Fluoroquinolone prophylaxis also lowers the incidence of FN and all-cause mortality following the first cycle of myelosuppressive chemotherapy for solid tumours. Levofloxacin has been the agent studied most thoroughly in this context. Although there is no convincing evidence that colonisation of individuals with resistant organisms due to antibiotic prophylaxis increases FN or mortality, such concerns must be taken seriously and the use of prophylaxis should be limited responsibly for patients with the greatest chance of benefit. Fluoroquinolone prophylaxis is well tolerated and cost-effective and should be offered to patients receiving chemotherapy for haematological malignancies and high-dose chemotherapy for solid tumours in which prolonged (>7 days) neutropenia is expected. It should also be considered for those receiving chemotherapy for solid tumours and lymphomas during the first cycle of chemotherapy when grade 4 neutropenia is anticipated.

For many years, controversy has surrounded the use of prophylactic antibiotics following chemotherapy for malignant diseases. Although effective, the toxicity of the trimethoprim–sulfamethoxazole combination led to a decline in its use, and raised questions about the prophylaxis of febrile neutropenia (FN) in general, particularly as mortality from FN was diminishing. However, as discussed elsewhere in this supplement (Cameron, 2009; Krell and Jones, 2009; Kelly and Wheatley, 2009; Jones and Leonard, 2009), data have emerged highlighting a range of risks associated with FN, including the adverse effects of consequent chemotherapy dose reduction and delays, FN morbidity, and mortality rates reaching 4–6%, as well as the costs of managing the condition (Trueman, 2009; Van de Wetering *et al*, 2005). Furthermore, some of the newer, effective chemotherapy agents, such as docetaxel and vinorelbine, are especially prone to causing FN (Aapro *et al*, 2006).

The fluoroquinolones, introduced in the 1980s, have transformed this field, becoming the most commonly used prophylactic antibacterial agents in neutropenic patients because of their broad antimicrobial spectrum, preservation of anaerobic gut flora (Walker, 1999), systemic bactericidal activity (Reeves, 1986), good tolerability and lack of myelosuppression (Del Favero and Menichetti, 1993).

In the past 5 years, major randomised trials and meta-analyses have led to significant progress in our understanding of the efficacy of fluoroquinolone prophylaxis, and the categories of patients (and chemotherapy regimens) associated with the greatest risk of FN, and hence those most likely to benefit from prophylactic treatment. The use of granulocyte colony-stimulating factors, with or without antibiotics, in the prophylaxis of FN is described in this supplement by Kelly and Wheatley. Our review summarises the evidence supporting the use of prophylactic antibiotics following chemotherapy and highlights the situations in which the gains are likely to be greatest.

## Recent research

Two large, investigator-led, randomised controlled trials published in 2005 provide firm evidence of the efficacy of fluoroquinolone prophylaxis in two distinct contexts: hospitalised patients expecting prolonged neutropenia, and patients receiving cyclical, mainly outpatient-based, chemotherapy causing neutropenia of a short duration.

### Hospitalised patients expecting prolonged neutropenia

Bucaneve *et al* (2005) reported findings from a double-blind, placebo-controlled trial of 760 hospitalised adult patients in whom chemotherapy-induced neutropenia (below 1000 neutrophils per mm^3^) was expected to last longer than 7 days. The trial included patients receiving chemotherapy for acute leukaemia, lymphomas or solid tumours. They were randomised to receive oral levofloxacin (500 mg daily) or placebo from the start of chemotherapy until the resolution of neutropenia. Intention-to-treat analysis showed a lower incidence of fever in patients receiving levofloxacin compared with the placebo group (65 *vs* 85%, respectively, *P*=0.001). Mortality was lower in the levofloxacin group but the study was not powered to prove this.

### Cyclical, mainly outpatient-based, chemotherapy causing neutropenia of short duration

In the UK Significant (simple investigation in neutropenic individuals of the frequency of infection after chemotherapy +/−antibiotic in a number of tumours) Trial (Cullen *et al*, 2005), 1565 patients receiving cyclical, mainly outpatient chemotherapy for solid tumours (predominantly breast, lung and testicular) or lymphoma, who were at risk of temporary, severe neutropenia (below 500 neutrophils per mm^3^) were randomised to receive either levofloxacin (500 mg daily) or placebo for 7 days during the expected neutropenic period in up to six cycles of chemotherapy. A significant reduction in febrile episodes and hospitalisation for treatment of bacterial infection was documented in the levofloxacin group during all cycles of treatment. Thirty-day mortality was lower in the levofloxacin group (1.5%) compared with the placebo group (2.3%), but the difference did not reach statistical significance (Cullen *et al*, 2005; Leibovici *et al*, 2006). The Significant Trial was by far the largest study looking specifically at antibiotic prophylaxis of FN in patients with solid tumours and lymphoma receiving moderately myelosuppressive chemotherapy, and it resolved the efficacy question for this group of patients.

## Meta-analyses examining mortality

As death from FN is relatively rare, meta-analyses are necessary to examine the effects of interventions on mortality. Gafter-Gvili *et al* (2005) undertook a meta-analysis of trials comparing prophylactic antibiotic therapy (fluoroquinolone-based and other regimens) with placebo or no intervention in patients receiving chemotherapy. They analysed 95 randomised controlled trials conducted between 1973 and 2004 involving 9283 patients. The primary outcome was all-cause mortality, and secondary outcomes included infection-related death, febrile episodes, bacteraemia, adverse events and emergence of bacterial resistance. The meta-analysis showed a statistically significant reduction in all-cause mortality of 34% in patients receiving prophylaxis compared with placebo or no intervention, and a 45% reduction in mortality in those receiving fluoroquinolones. Although the relative risk of death did not differ between haematological malignancies and solid tumours in this meta-analysis, the number of solid tumours was much smaller. Consequently, the meta-analysis has been updated (Leibovici *et al*, 2006) to include data from GIMEMA (Gruppo Italiano Malattie Ematologiche Maligne dell'Adulto) (Bucaneve *et al*, 2005) and the Significant Trial (Cullen *et al*, 2005). Among patients with acute leukaemia, who had undergone bone marrow transplantation, the relative risk of death with fluoroquinolone prophylaxis was 0.67 (0.55–0.83) – a one-third reduction compared with the control group, which did not receive prophylaxis. Among patients with solid tumours and lymphomas, fluoroquinolone prophylaxis had a significant impact on all-cause mortality during the first cycle of chemotherapy, with a relative risk of 0.48 (0.26–0.88), compared with controls.

## Variables that affect fn risk and prophylactic efficacy

The effect of cycle number on the risk of FN has been known but under-appreciated for some years. Studies in small-cell lung cancer and breast cancer have shown that the risk of FN is much greater following the first cycle of chemotherapy compared with later cycles (Holmes *et al*, 2002; Timmer-Bonte *et al*, 2005; Vogel *et al*, 2005). This finding has been confirmed in surveys of larger numbers of patients with lymphoma (Lyman and Delgado, 2003) and multiple tumour types (Crawford *et al*, 2004). There are several possible explanations for the first-cycle effect. For example, neutropenia that is not accurately predictable for a given patient may be severe in the first cycle, then reduced when subsequent cycles are subject to secondary modification, such as dose reduction. Alternatively, the cytoreductive effects of the first chemotherapy cycle may enable resolution of a cancer-related focus of infection (e.g., beyond an obstructed airway in a patient with lung cancer) or lead to an improvement in performance status. Other variables that predict increased rate of FN are discussed elsewhere in this supplement (Kelly and Wheatley, 2009).

## Rational selection of patients for antibacterial prophylaxis

**Figure 1:**
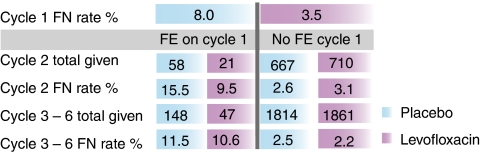
FN rate per cycle and impact on later events in the Significant Trial (Cullen *et al*, 2007). FE, Febrile episode.

A second publication from the Significant Trial examined chemotherapy cycle effects and other variables that might predict increased efficacy of levofloxacin prophylaxis (Cullen *et al*, 2007). It showed that the incidence of FN was 8% in first cycles but only 3.3% per cycle thereafter. In addition, prophylaxis was more effective in first cycles (odds ratio 0.42, *P*<0.001) than in later cycles (odds ratio 0.78). However, FN in cycle 1 predicted a much higher risk of subsequent FN and a trend towards continued prophylactic efficacy in later cycles (Figure 1). Among the cancers studied, the rate of FN was greatest for testicular cancer (27.9%), followed by small-cell lung cancer (17.3%), and lowest for breast cancer (11.5%). Prophylactic efficacy was consistent despite differences in age, sex, performance status, treatment context (adjuvant or advanced) and disease type (except possibly non-Hodgkin's lymphoma).

In the light of the pressure to limit antibacterial use (for reasons discussed below), the data on cycle effects support the practice of offering prophylactic levofloxacin in the first cycle of myelosuppressive cancer chemotherapy, and in subsequent cycles only if there has been a fever in cycle 1. These data also show that prophylactic levofloxacin is effective regardless of the patient's age or performance status, or the type of solid tumour (Cullen *et al*, 2007).

## Concerns about antibiotic prophylaxis

### Treatment cost

Bucaneve *et al* (2005) showed that five patients undergoing chemotherapy for cancer needed to be treated with oral levofloxacin to prevent one episode of FN. The average length of prophylaxis in the study was 14 days for patients receiving chemotherapy for solid tumour or lymphoma, and 27 days for patients with acute leukaemia. A 7-day course of levofloxacin in the UK costs £18.10 (BNF, 2009). It therefore costs only £181.00 and £349.07, respectively, to prevent an episode of FN in these two groups. The Significant Trial did not directly address the economic aspects of prophylaxis. However, analysis shows that 23 patients needed to be treated with levofloxacin to prevent one episode of FN (Table 1), and that the cost of prophylaxis for 23 patients for one cycle of chemotherapy is approximately £416.30 (Cullen *et al*, 2005). The cost of managing one episode of FN in the UK has been estimated as £4064.84 (Holmes *et al*, 2004), suggesting that antibiotic prophylaxis is cost-effective in these patient groups.

### Antibiotic resistance

The main concern over the use of prophylactic antibiotics remains the emergence of antibiotic resistance, and its implications both for the individual patient and at ward level.

There is no doubt that routine prophylactic use of antibiotics can cause colonisation of individual patients with resistant organisms, but the clinical relevance of this is unclear. Bucaneve *et al* (2005) observed a non-significant increase in the incidence of levofloxacin-resistant Gram-negative bacteraemia among patients receiving levofloxacin, but this did not affect outcomes such as infection-related morbidity or mortality. Gafter-Gvili *et al* (2005) found that the risk of developing fluoroquinolone resistance did not increase significantly secondary to prophylaxis, and that there was a low incidence of infections caused by resistant bacteria in patients who had received prophylaxis.

There have been several reports of the emergence of fluoroquinolone-resistant bacteria in units that practise fluoroquinolone-based prophylaxis (Razonable *et al*, 2002; Kern *et al*, 2005). However, there is no convincing evidence that patients have suffered adverse outcomes as a result. Kern *et al* (2005) found that after fluoroquinolone prophylaxis had been in use for 10 years, there was an increase in the number of cancer patients colonised or infected with fluoroquinolone-resistant *Escherichia coli*. The practice of prophylaxis was stopped for 6 months in the unit, and a significant increase in the incidence of Gram-negative bacteraemia was found in patients with cancer, accompanied by a decrease in the proportion of fluoroquinolone resistance in *E coli* bacteraemia. After the resumption of prophylaxis, an increase in the proportion of *in vitro* fluoroquinolone resistance in *E coli* bacteraemia was observed, but the incidence of all Gram-negative bacteraemia was reduced to pre-discontinuation levels. The authors suggest that the rate of resistance in their unit is a poor indicator of the potential clinical benefits associated with fluoroquinolone prophylaxis in patients with cancer.

## NCCN guidelines

In 2008, the US National Comprehensive Cancer Network published guidelines on the prevention and treatment of cancer-related infections (Segal *et al*, 2008). It recommends prophylactic fluoroquinolones for high-risk and intermediate-risk groups, which largely comprise patients receiving high-dose chemotherapy and those with haematological malignancy in which the anticipated duration of neutropenia is longer than 7 days. For most solid tumours undergoing standard outpatient cyclical chemotherapy, in which the anticipated duration of neutropenia is less than 7 days, prophylactic fluoroquinolones are not recommended, because of the risk of microbial resistance. However, even in the latter circumstances, we believe that when grade 4 neutropenia is expected (e.g., etoposide-containing regimens for testicular and small-cell lung cancers, and regimens containing docetaxel, vinorelbine or doxorubicin), in which the risk of FN is very high, fluoroquinolones should be considered, particularly in the first cycle.

## Conclusion

There is now convincing evidence that antibiotic prophylaxis reduces the incidence of FN and mortality in patients receiving cytotoxic chemotherapy for acute leukaemia and for patients with solid tumours and lymphoma receiving high-dose chemotherapy (Segal *et al*, 2008). Therefore, we would argue that antibiotic prophylaxis should be offered routinely to these groups of patients.

Fluoroquinolone prophylaxis also significantly reduces FN in patients with solid tumours or lymphoma who are undergoing cyclical standard-dose myelosuppressive chemotherapy (Cullen *et al*, 2005). A significant impact on all-cause 30-day mortality was also shown in this group (Leibovici *et al*, 2006). We believe prophylaxis is indicated during the first cycle of chemotherapy in which there is an expectation of grade 4 neutropenia (below 500 neutrophils per mm^3^).

Fluoroquinolones are the most effective agents for prophylaxis of FN, and are cost effective and well tolerated (Reeves, 1986; Del Favero and Menichetti, 1993; Walker, 1999). When choosing between the fluoroquinolones, clinicians should take into account the patterns of pathogens and resistance in their patient population, and remember that, compared with ciprofloxacin, levofloxacin has additional activity against Gram-positive organisms but less anti-pseudomonal activity (MacGowan *et al*, 1999; Montanari *et al*, 1999). Compliance is a major concern when considering oral prophylactic therapy, so once-daily levofloxacin may have an advantage in this regard.

The main concern relating to the prophylactic use of antibiotics remains the development of resistance. Although it is established that fluoroquinolone prophylaxis can result in increased fluoroquinolone resistance in treatment centres, there is little evidence of a resultant increase in FN or infection-related mortality (Bucaneve *et al*, 2005).

There are also important ethical concerns about withholding a proven treatment from current patients for the sake of an unquantified benefit to patients in the future.

## Figures and Tables

**Table 1:** Levofloxacin *vs* placebo to prevent infection after chemotherapy in patients with solid tumours or lymphoma (Cullen *et al*, 2005)

**Outcomes**	**Levofloxacin (%)**	**Placebo (%)**	**RRR (95% CI)**	***P*-value**	**NNT (CI)**
*In first cycle*					
Febrile episode	3.5	7.9	56% (32–72)	<0.001	23 (15–46)
Probable infection	14	19	28% (10–43)		19 (11–58)
Hospitalisation	6.7	10	36% (10–54)		28 (16–109)
					
*In any cycle*					
Febrile episode	11	15	29% (8.1–45)	0.01	23 (13–91)
Probable infection	34	41	18% (6.3–27)		14 (9–41)
Hospitalisation	16	22	27% (9.9–41)	0.004	18 (11–52)
Severe infection or death	1.0	2.0	50% (-14–78)	NS	

RRR=Relative risk reduction; NNT=Number needed to treat; NS=Not significant.
